# Impact of body mass index on blood pressure and cardiovascular adaptation to isometric exercise training 

**DOI:** 10.1007/s00421-026-06248-z

**Published:** 2026-04-28

**Authors:** Harry T. Swift, Christopher K. Farmer, Jonathan D. Wiles

**Affiliations:** 1 School of Psychology and Life Sciences, Canterbury Christ Church University, Kent, CT1 1QU UK; 2 Department of AI and Preventative Medicine, Kings College London, London, WC2R 2LS UK; 3 Centre for Health Services Studies, University of Kent, Canterbury, Kent, CT2 7PE UK

**Keywords:** Blood pressure, Total peripheral resistance, Heart rate variability, Body mass index, Obesity, Overweight

## Abstract

**Objectives:**

Obesity is characterised by excess adipose tissue, which impairs vascular function and blood pressure (BP) regulation. However, it remains unclear whether adiposity influences the BP-lowering response to isometric exercise training (IET). This randomised-controlled trial explored the impact of body mass index on BP and cardiovascular adaptation following IET.

**Methods:**

80 participants with normal to high-normal systolic BP (range 120-140 mmHg) were recruited for the study. Participants were randomised into two BMI groups: normal BMI (18.5–24.9 kg/m²; n=40) and high BMI (25-34.9 kg/m²; n=40) and then either performed home-based lower-body IET, 3 times per week, for 4 weeks (each session comprised 4 x 2-minute bouts), or were allocated to the control group. Cardiovascular variables, including BP, total peripheral resistance (TPR), and heart rate variability (HRV), were measured at rest pre- and post-intervention.

**Results:**

Overall, there were significant reductions in systolic BP, diastolic BP and mean arterial pressure (MAP) in the IET group compared to control. However, the normal-BMI group had significantly greater reductions in diastolic BP (– 8.5 [– 11.8, – 5.3] mmHg) and MAP (– 9.2 [– 11.9, – 6.6] mmHg) compared to the high-BMI group (dBP: – 2.6 [– 6.2, 1.1]; MAP: -4.7 [– 7.6, – 1.8]). Although TPR was significantly greater in the high-BMI group at week 4 (p=0.022), it was also elevated at baseline, and change-score analyses revealed no significant between-group difference in the magnitude of TPR reduction (Δ p=0.964).

**Conclusion:**

These findings indicate that BMI may modulate the BP-lowering response to IET, suggesting that individuals with higher BMI may require adjunctive or prolonged interventions to optimise BP reduction.

## Introduction

Obesity is a complex multifactorial disease and a pressing public health issue globally (Powell-Wiley et al. 2021). Obesity is strongly associated with a range of comorbidities, including hypertension, insulin resistance, and dyslipidaemia, which collectively increase the risk of cardiovascular disease and associated morbidity and mortality (Blüher 2019; Powell-Wiley et al. 2021). In 2022, 2.5 billion adults globally (43%) were overweight and 890 million (16%) were obese, more than double the burden since 1990 (WHO 2024). Assuming the continuation of historical trends, by 2050, it is forecasted that the total number of adults either overweight or living with obesity will reach 3.8 billion, which is over half of the likely global adult population at that time (Ng et al. 2025).

The expansion and remodelling of adipose tissue as a result of obesity affects multiple adipose depots and plays a significant role in vascular dysfunction, contributing to increased vascular resistance and elevated blood pressure (BP) (Bunsawat et al. 2021; Koenen et al. 2021). Additionally, obesity is associated with autonomic nervous system imbalance, characterised by heightened resting sympathetic, and reduced parasympathetic tone, both of which promote vasoconstriction and sustained elevations in vascular tone (Espinoza-Salinas et al. 2021), contributing to increases in BP (Valensi 2021). Indeed, hypertension is among the most prevalent cardiometabolic complications observed in individuals with obesity (DeMarco et al. 2014). Compounding this issue, obesity is associated with treatment‑resistant hypertension and altered drug handling (Gouju and Legeay 2023), meaning that multiple antihypertensive agents are often required and some classes (notably traditional β‑blockers and, to a lesser extent, thiazide diuretics) may worsen associated comorbidities such as insulin resistance and dyslipidaemia, or promote further weight gain (Shariq and McKenzie 2020; Parvanova et al. 2025). This underscores the importance of effective non‑pharmacological interventions.

Physical activity is widely recognised as a first-line non-pharmacological intervention for managing elevated BP (McCarthy et al. 2025). However, the BP-lowering effects of exercise may be attenuated in individuals with obesity. Aerobic exercise interventions are associated with relatively modest reductions in BP amongst individuals with obesity, typically in the region of ~ 3.5 mmHg for sBP and ~ 2.0 mmHg for diastolic BP (dBP). In contrast, interventions that combine exercise with dietary-induced weight loss produce substantially larger reductions in BP (sBP: -12.5 mmHg; dBP: -7.9 mmHg) (Bacon et al. 2004). These findings suggest that adiposity may attenuate the BP-lowering effects of exercise when performed in isolation, and that reductions in fat mass could play a critical role in maximising cardiovascular adaptations to lifestyle intervention (Bacon et al. 2004).

Isometric exercise training (IET) has emerged as one of the most efficacious exercise modalities for lowering resting BP, with recent meta-analytic evidence showing greater reductions than aerobic, dynamic resistance and concurrent training methods (Edwards et al. 2023). The magnitude of BP reduction following IET also equals or exceeds that achieved through antihypertensive medications (Law et al. 2009; Paz et al. 2016) or dietary weight loss alone (Bacon et al. 2004). Despite this growing body of evidence, limited research has explored the potential moderating role of body mass index (BMI) on BP outcomes following IET. Some studies have suggested that BP reductions may be more pronounced in individuals with normal BMI compared to those with high BMI (Loaiza-Betancur and Chulvi-Medrano 2020), although considerable heterogeneity in effect size and methodological differences limit the confidence of these findings. A recent multilevel meta‑review in pre‑ to established hypertension also reported that BMI moderated dBP change, but not sBP, with overweight and obese individuals showing an attenuated response in dBP following IET (Zhou et al. 2025). Moreover, Baross et al. (2013) reported significant reductions in sBP (11 ± 8 mmHg), but not dBP, following 4 weeks of IET in individuals with high BMI (27.9 ± 2.3 kg/m²) and suggested that higher adiposity may attenuate diastolic adaptations to a greater extent than systolic. Conversely, Smart et al. (2019) found no significant relationship between BMI and BP reduction following IET and suggested that a high BMI does not attenuate the effectiveness of IET. Collectively, these findings highlight that the influence of BMI on IET response remains unclear and requires further research.

Understanding whether BMI modulates the effectiveness of IET on BP reduction is essential for optimising clinical exercise prescriptions. Identifying whether individuals with high BMI exhibit differential responses to IET than their normal-BMI counterparts could inform personalised hypertension management strategies and improve the scalability of IET as a public health tool. It could also highlight the need for individualised and well-controlled applications in research settings.

Therefore, this study aimed to engage both normal and high-BMI participants with an established IET protocol compared to control, and then (1) explore any differences in the BP adaptations between normal and high BMI following IET and (2) investigate any differences in the associated physiological mechanisms between the BMI categories. It was hypothesised that individuals with high BMI would exhibit inferior reductions in resting BP following IET compared to their normal-BMI counterparts.

## Methods

### Study population

Both male and female participants with normal to high-normal sBP (range 120–140 mmHg) and a total weekly physical activity below 150 min of moderate activity (≤ 600 MET-minutes/week based on light-intensity activities only) were recruited for the study. Participants were categorised into two BMI groups for descriptive and analytical purposes: a normal BMI group (BMI 18.5–24.9 kg/m²) and a high BMI group (BMI 25–34.9 kg/m²). BMI was calculated as body mass (kg) divided by height squared (m²), and classifications followed WHO criteria (WHO 2024). Overweight (BMI 25.0–29.9 kg/m²) and obese class I (BMI 30.0–34.9 kg/m²) individuals were combined into a single high-BMI group a priori as the study was designed to detect a binary contrast between normal and elevated adiposity rather than to characterise dose-response relationships across BMI subgroups. Individuals with a low BMI < 18.5 kg/m² or a very-high BMI ≥ 35 kg/m² (obesity class II or above) were excluded from this study.

Participants were also excluded if they reported any structured exercise ≥ 4 METs for ≥ 10 min, had injuries or any significant comorbidities, were taking or had previously taken anti-hypertensive medication, were smokers, or consumed more than 14 units of alcohol per week. Screening was conducted using a standard Physical Activity Readiness Questionnaire. All participants provided written informed consent. The procedures conformed to the Declaration of Helsinki and the study was approved by the ethics committee of Canterbury Christ Church University (Ref: 19/SAS/12 C).

### Study design

The required sample size (*n* = 80) was calculated using G*Power (Heinrich-Heine-Universität Düsseldorf, Düsseldorf, Germany) (Faul et al. 2009). For a direct comparison of the primary outcome variable (sBP) between the BMI categories, based on an alpha level of 0.05, a power of 0.80, two groups (normal BMI and high BMI), and one covariate (baseline BP values), a sample size of 60 participants (30:30) was able to detect a small-to-moderate effect (0.37 Cohens f). For the first phase of the study comparing all 60 intervention participants (normal and high BMI) to a control group of 20 participants, the estimated power based on likely changes observed by Taylor et al. (2019) was in excess of 95% for both sBP and dBP.

Participants were recruited using a quota-based strategy to ensure balanced representation of normal and high-BMI categories across study arms. Recruitment targets were set a priori to achieve equal representation within the intervention group and comparable representation within the control group. Allocation to intervention or control within each BMI category was performed using a computer-generated randomisation procedure (RAND function, Microsoft Excel). Owing to the nature of the exercise intervention, blinding of participants and researchers was not feasible.

There was a total of four visits to the laboratory for all intervention participants. Upon arrival to the laboratory at the first visit, a seated resting BP was performed using an automated BP monitor (Dinamap, PRO 200, GE Medical Systems Information Technologies GmbH, Munzinger Strasse 3, 79111, Freiburg, Germany) to ascertain the participants resting BP. BP was measured three times on the left arm at 5-min intervals after a 15-min period of quiet seated rest. Individuals were excluded from the study if they were hypertensive, as defined by having a resting BP of SBP ≥ 140 mm Hg or a DBP ≥ 90 mm Hg (Mancia et al. 2023). Participant stature (cm) using a stadiometer (Seca 213, Seca GmbH & Co. Kg., Hamburg, Germany) and body mass (kg) using mechanical column scales (Seca 710, Seca GmbH & Co. Kg, Hamburg, Germany) were also measured during this first visit to ascertain BMI status. Prior to the commencement of BP measurements, participants were familiarised with the equipment and BP measurement procedures. Participants were then invited for a second visit to complete an incremental wall squat test to determine their individualised knee joint angle used during training, as previously described by Wiles et al. (2017). The third and fourth visits involved recording physiological data before and after the 4-week training period, as outlined below. Height and body mass were also measured during the third and fourth visit to ensure that there were no significant changes in BMI that could influence the primary outcome variables. Control participants were requested to attend the laboratory three times; a familiarisation session, baseline resting measures and week-4 resting measures.

Both intervention and control participants were asked to maintain habitual dietary habits and daily routines throughout the study. Participants were asked to attend the laboratory at the same time of the day (± 2 h), fast for 4 h and avoid caffeine and alcohol for 24 h before testing. For intervention participants, post IET measures at week 4 were taken between 72 and 96 h after the participant’s final training session to avoid the influence of acute hypotension that may persist post-exercise.

### Resting measures

All haemodynamic and cardiac autonomic variables were measured using a validated Task Force Monitor (TFM) (CNSystems, Graz, Austria) (Fortin et al. 2001). All measurements were taken in a temperature-controlled room (20 ± 1 degree C) pre- and post-IET. To measure continuous BP, the TFM uses a vascular unloading technique at the proximal limb of the index or middle finger (Parati et al. 2003). Continuous measurement of BP is then adjusted in accordance with oscillometric BP values from the brachial artery of the contralateral arm (Gratze et al. 1998). Heart rate (HR) was recorded using a 6-channel ECG. Beat-to-beat stroke volume (SV) was measured with impedance cardiography via one electrode band applied to the nape of the neck and two placed on either side of the thorax, in line with the xiphisternum (Gratze et al. 1998). Q̇ is then subsequently calculated as the product of HR and SV and TPR is measured according to Ohm’s law.

To measure R-R intervals, a 6-channel ECG was used, with the subsequent beat-to-beat values used to calculate real-time HRV via an autoregressive model (Fortin et al. 2001). The ECG traces were manually screened to clear any erroneous data. High (HF) (predominantly parasympathetic outflow) and low (LF) (predominantly sympathetic outflow) (Kim et al. 2018) frequency parameters of HRV were automatically calculated by the TFM and expressed in absolute units (ms^2^). Baroreceptor reflex sensitivity (BRS) was automatically calculated via the sequence method (Fortin et al. 2001).

Resting haemodynamic and cardiac autonomic measures were measured at baseline and at 4 weeks. Following 15-minutes seated rest, haemodynamic and cardiac autonomic function was measured continuously in the seated position for 5 min. After this time, the mean for each variable was calculated offline for the 5-minute period.

### Isometric exercise training intervention

The exercise training intervention consisted of a previously validated 4-week lower body wall squat IET programme (Wiles et al. 2017) three times per week over a 4-week period, with 48 h between each session. Each training session comprised of four bouts of IET exercise separated by 2 min of seated res. The training was performed at a participant-specific knee joint angle ascertained during an incremental IET test, which was relative to 95% HR_peak_. Participants were required to record their HR during the last 30 s of each exercise bout using a wrist-mounted HR monitor and a Polar H10 HR chest strap (Polar Electro Oy). If the HR data fell outside the target heart rate zone for more than two sessions, the knee angle was reduced or increased by 5° to ensure that the HR fell back within the correct range. This data was also used to confirm adherence to training.

### Data analysis

**Fig. 1:**
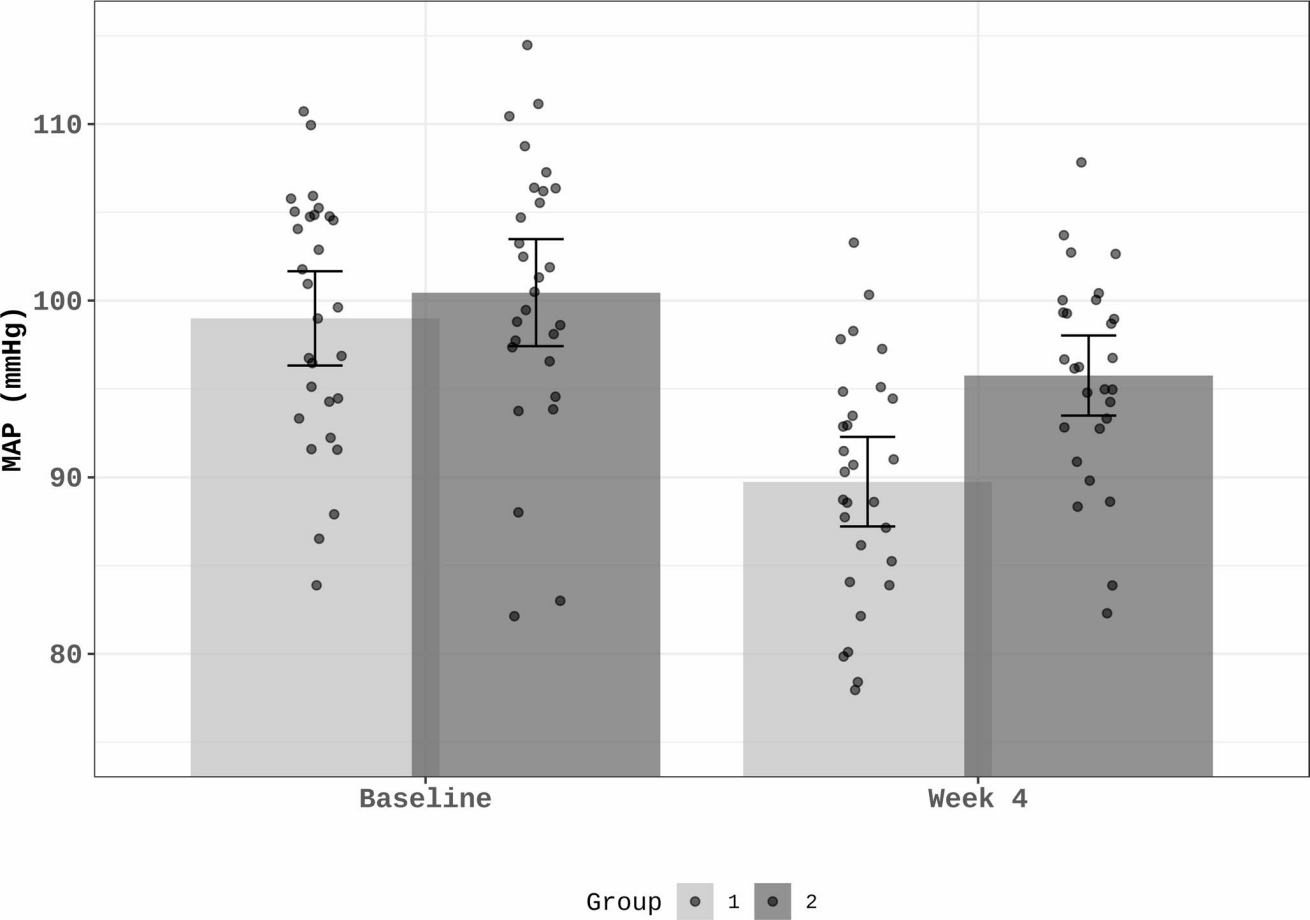
Mean MAP at baseline and week 4 in Group 1 (normal BMI) and Group 2 (high BMI). Bars represent group means and error bars indicate 95% confidence intervals. Individual data points are shown to illustrate within-group variability. MAP is expressed in mmHg

**Fig. 2:**
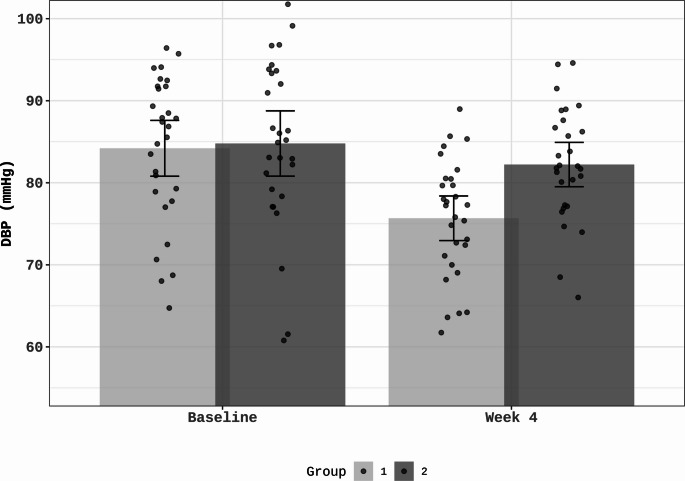
Mean dBP at baseline and week 4 in Group 1 (normal BMI) and Group 2 (high BMI). Bars represent group means and error bars indicate 95% confidence intervals. Individual data points are shown to illustrate within-group variability. dBP is expressed in mmHg

**Fig. 3:**
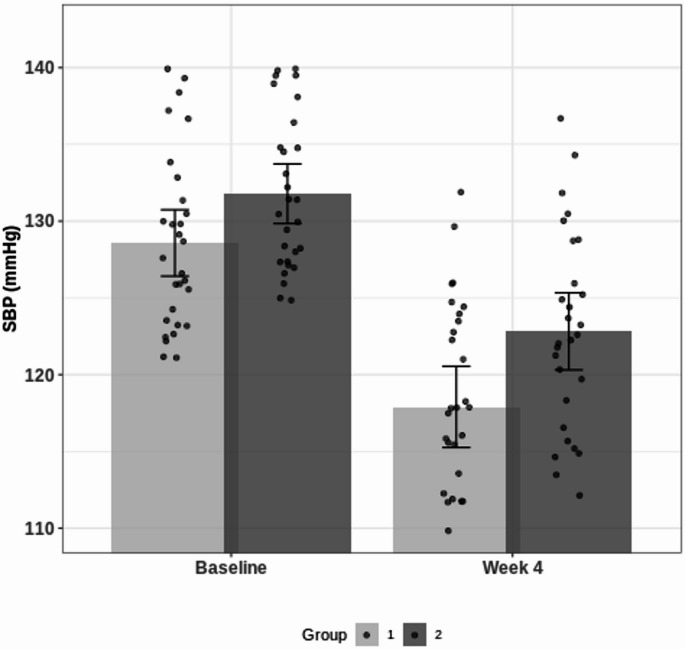
Mean sBP at baseline and week 4 in Group 1 (normal BMI) and Group 2 (high BMI). Bars represent group means and error bars indicate 95% confidence intervals. Individual data points are displayed to show the distribution of participant-level values. sBP is expressed in mmHg

Data were checked for normality assumptions. Where these were met, a one-way analysis of covariance (ANCOVA) was first carried out to explore the overall differences between all intervention participants (*n* = 60) compared to a control group (*n* = 20), with baseline measures as the covariate. Differences in each dependent variable (week-4 resting measures) between normal-BMI (*n* = 30) and high-BMI (*n* = 30) participants were then explored using a one-way ANCOVA with baseline measures as the covariate. These analyses were conducted separately rather than within a single two-way model. The control group was intentionally kept small (*n* = 20), as the primary aim of the study was not to establish the efficacy of IET against control, but rather to examine whether BMI moderates the BP response to training. Where normality assumptions were violated, a Quade test was applied with the grouping variable as the treatment factor and ranked baseline values as the blocking variable. Change scores were calculated as the difference between week-4 and baseline values to describe the magnitude of improvement within each group and to support interpretation of the primary results. Effect sizes were calculated as Cohen’s d, with values of 0.2, 0.5, and 0.8 interpreted as small, medium, and large respectively. 95% confidence intervals are reported for all change scores. To examine whether a quantitative relationship existed between BMI and BP responses to training, Pearson correlation coefficients were calculated between BMI and change scores (week 4 - baseline) for all BP variables. An alpha level of < 0.05 was set as the threshold for statistical significance (Figs. 1, 2 and 3).

## Results

77 participants were included in the final analysis. The study experienced an 4% attrition rate, with 3 participants lost to follow-up (all within the intervention groups). This included 2 withdrawn participants for non-compliance with the protocol, and 1 voluntarily withdrawal. No adverse events were reported during the study. Of the 29 normal-BMI participants allocated to the IET intervention, 1 missed a single session (99% adherence). Of the 28 high-BMI participants allocated to the IET intervention, 1 missed a single session (99% adherence) (Table 1).

**Table 1:** Baseline characteristics of all participants.

Variable	Normal-BMI IET (*n* = 29)	High-BMI IET (*n* = 28)	Normal-BMI CON (*n* = 10)	High-BMI CON (*n* = 10)
sBP/dBP	129/84	132/85	127/81	128/81
Age (years)	37 ± 11	35 ± 9	33 ± 12	36 ± 8
Height (cm)	175 ± 10*	170 ± 8*	172 ± 7	178 ± 9
Body mass (kg)	70 ± 8*	90 ± 13*	66 ± 8	89 ± 11
BMI	23 ± 2*	31 ± 3*	22 ± 2	28 ± 4
Sex	M:14, F:15	M:15, F:13	M:5, F:5	M:7, F:3

Baseline sBP/dBP, age, height, body mass, mean BMI and sex for all participants at baseline; * = significant difference between groups (*p* < 0.05).

There were statistically significant differences between the IET groups in height (*p* = 0.045), body mass (*p* < 0.001) and BMI (*p* < 0.001).

### Overall blood pressure effect compared to control

Following the 4-week training period, resting sBP (M ± SD) decreased in the intervention group (− 9.8 ± 6.7 mmHg) and was significantly different from the control group (− 0.1 ± 4.0 mmHg; *p* < 0.001). Resting dBP was also reduced following the intervention (− 5.6 ± 9.9 mmHg) and significantly differed from the control group (2.8 ± 4.6 mmHg; *p* = 0.003). Similarly, reductions in MAP following the intervention (− 7.0 ± 7.8 mmHg) were significantly greater than those observed in the control group (2.6 ± 3.8 mmHg; *p* < 0.001).

### Between group blood pressure responses

Between-group analyses revealed significant differences in BP responses to IET based on BMI classification. Individuals with a normal BMI experienced significantly greater reductions in dBP (*p* < 0.001) (Fig. 2) and MAP (*p* < 0.001), (Fig. 1) but not sBP (*p* = 0.47) (Fig. 3), compared with those with high BMI. Change-score analyses supported these findings: reductions in dBP (Δ = − 6.0 mmHg, *p* = 0.015) and MAP (Δ = − 4.5 mmHg, *p* = 0.021) were significantly larger in the normal-BMI group, while sBP change did not differ between groups (Δ = − 1.7 mmHg, *p* = 0.367).

### BP and BMI correlation analysis

BMI was not significantly correlated with ΔsBP (*r* = 0.09, *p* = 0.516). Small but statistically significant positive correlations were observed between BMI and ΔdBP (*r* = 0.29, *p* = 0.029) and ΔMAP (*r* = 0.27, *p* = 0.044).

### Haemodynamic and cardiac autonomic adaptations

There were significant between-group differences for TPR (*p* = 0.022), with the high-BMI group exhibiting higher TPR at week 4. However, change-score analyses showed no significant group differences in the magnitude of improvement for TPR (Δ *p* = 0.964). All other haemodynamic and cardiac autonomic variables showed no significant between-group differences in either ANCOVA or change-score analyses (*p* > 0.05) (Table 2).

**Table 2:** Resting blood pressure, haemodynamic and cardiac autonomic variables at baseline and week 4 in normal-BMI and high-BMI intervention groups, with within-group mean changes (95% CI) and effect sizes.

Variable	Normal-BMI BL (mean ± SD)	High-BMI BL (mean ± SD)	Normal-BMI Week 4 (mean ± SD)	High-BMI Week 4 (mean ± SD)	Normal-BMI Change (mean [95% CI])	Effect Size (d)	High-BMI Change (mean [95% CI])	Effect Size (d)
HR	73.7 ± 9.0	72.5 ± 10.8	70.2 ± 10.5	71.0 ± 10.8	– 3.4 [– 7.6, 0.8]	0.31	– 1.6 [– 5.7, 2.4]	0.15
sBP	128.6 ± 5.7	131.8 ± 5.0	117.9 ± 7.0	122.8 ± 6.5	– 10.7 [– 13.4, – 8.0]	1.49	– 9.0 [– 11.7, – 6.2]	1.28
dBP	84.2 ± 9.0	84.8 ± 10.3	75.7 ± 7.2	82.2 ± 7.0	– 8.5 [– 11.8, – 5.3]	1.0	– 2.6 [– 6.2, 1.1]	0.27
MAP	99.0 ± 7.0	100.4 ± 7.8	89.8 ± 6.6	95.8 ± 5.9	– 9.2 [– 11.9, – 6.6]	1.34	– 4.7 [– 7.6, – 1.8]	0.62
SV	85.5 ± 17.7	78.5 ± 26.9	84.5 ± 23.0	76.4 ± 15.9	– 1.0 [– 8.5, 6.6]	0.05	– 2.1 [– 12.0, 7.7]	0.08
SVi	46.5 ± 10.3	38.9 ± 13.1	45.8 ± 11.8	38.1 ± 8.4	– 0.7 [– 4.9, 3.5]	0.06	– 0.9 [– 5.7, 3.9]	0.07
CO	6.3 ± 1.4	5.7 ± 2.0	5.8 ± 1.2	5.4 ± 1.2	– 0.5 [– 1.0, 0.1]	0.31	– 0.3 [– 1.0, 0.4]	0.18
Ci	3.4 ± 0.8	2.8 ± 1.0	3.2 ± 0.7	2.7 ± 0.6	– 0.3 [– 0.6, 0.1]	0.31	– 0.2 [– 0.5, 0.2]	0.18
TPR	1298.9 ± 374.6	1585.5 ± 482.9	1097.8 ± 279.2	1388.8 ± 352.0	– 201.1 [– 321.1, -81.2]	0.64	– 196.7 [– 361.9, – 31.5]	0.46
TPRi	708.8 ± 220.0	792.4 ± 262.1	600.2 ± 170.4	694.9 ± 201.8	– 108.7 [– 175.3, – 42.1]	0.62	– 97.5 [– 179.7, – 15.4]	0.46
LF	1025.3 ± 947.0	1096.0 ± 1112.8	1016.1 ± 779.1	1270.0 ± 953.1	– 9.2 [– 247.6, 229.3]	0.01	174.0 [– 165.2, 513.2]	0.2
HF	569.4 ± 489.1	417.9 ± 470.5	856.7 ± 716.8	969.1 ± 997.9	287.3 [34.8, 539.7]	0.43	551.2 [158.6, 943.8]	0.54
LF: HF	2.8 ± 2.2	3.4 ± 2.2	1.7 ± 1.0	2.2 ± 2.0	– 1.1 [– 2.0, – 0.3]	0.5	– 1.2 [– 2.2, – 0.1]	0.43
PSD-RRI	2154.0 ± 1313.2	2028.3 ± 1569.9	2864.7 ± 1517.4	2710.1 ± 1748.6	710.7 [162.6, 1258.8]	0.49	681.8 [78.6, 1285.0]	0.44
BRS	13.3 ± 6.1	12.5 ± 6.8	23.0 ± 12.5	21.0 ± 13.1	9.7 [4.4, 14.9]	0.7	8.2 [4.1, 12.3]	0.77

Mean values and SD for resting heart rate (HR bpm), total peripheral resistance (TPR dyne/s/cm5), TPR index (TPRi dyne/s/m2/cm5), cardiac output (Q̇ l/min), cardiac output index (Q̇I l/min / m2), stroke volume (SV ml) and SV index (SI ml/m2) before, during and after IET for the normal and high-BMI intervention groups.

## Discussion

The primary aim of this study was to explore any differences in BP reduction and associated cardiovascular mechanisms between participants with normal and high BMI following 4 weeks of IET. When analysing all participants combined (normal and high-BMI) there was a significant reduction in resting BP compared with the control. This finding is in agreement with previous studies utilising lower-body wall-squat IET (Wiles et al. 2017; Taylor et al. 2019; Decaux et al. 2022; Cohen et al. 2023; Lea et al. 2023). However, there were significant differences in the magnitude of BP reduction between BMI categories. Participants with normal BMI (18.5–24.9 kg/m²) exhibited significantly greater reductions in dBP and MAP compared with those with high BMI (25–34.9 kg/m²). Systolic BP did not differ significantly between groups. Small but significant positive correlations were also found for ΔdBP and ΔMAP, but not for ΔsBP, suggesting a modest quantitative relationship between BMI and BP responsiveness to IET, consistent with the group-level findings. Overall these data indicate an attenuation of BP responsiveness to IET in individuals with higher BMI.

These findings extend previous work (Zhou et al. 2025; Loaiza-Betancur and Chulvi-Medrano 2020; Baross et al. 2013), showing that higher BMI may dampen BP reductions. Notably, dBP dynamics are more strongly influenced by peripheral vascular resistance and small-artery structure than sBP, and the diminished improvements in dBP in the high BMI group aligns with known obesity-related impairments in vascular function (Kwaifa et al. 2020). Obesity is associated with a constellation of vascular abnormalities driven by adipose tissue expansion and dysfunction. Chronic low-grade inflammation, characterised by increased IL-6, TNF-α, MCP-1 and leptin, alongside reduced adiponectin, combined with expansion of visceral and perivascular adipose tissue, shifts adipokine signalling toward greater secretion of vasoconstrictive mediators (e.g., endothelin-1, angiotensin II, resistin) (Kwaifa et al. 2020). This transition drives perivascular adipose tissue from an anti- to a pro-contractile phenotype, providing a mechanistic basis for the blunted dBP responsiveness observed in the high-BMI group (Kwaifa et al. 2020; Młynarska et al. 2025; Koenen et al. 2021). Furthermore, obesity-related insulin resistance is closely linked to endothelial dysfunction and increased arterial stiffness (Hill et al. 2021). In normal-weight individuals, insulin typically stimulates endothelial nitric oxide (NO) production to promote vasodilation (Kuboki et al. 2000). In obesity, this NO-mediated signalling is blunted due to endothelial insulin resistance and heightened oxidative stress, limiting vascular adaptability, increasing peripheral resistance, and thereby constraining the potential for reductions in BP following exercise training (Koenen et al. 2021; Paneni 2015).

Adipose tissue expansion is also accompanied by inadequate angiogenesis and microvascular rarefaction (Crewe et al. 2017), which leads to local hypoxia, inflammation, endothelial dysfunction, and impaired vasodilatory capacity, all of which have been shown to contribute to elevated peripheral vascular resistance (Bunsawat et al. 2021; Koenen et al. 2021). In the current study there were significant between-group differences for TPR found at week 4 which may also help to explain the differences in the dBP reductions seen. Indeed, meta-analytic evidence indicates that reductions in TPR are the primary haemodynamic driver of BP changes following IET (Edwards et al. 2022). However, these differences were not supported by change-score analyses, indicating they reflected cardiovascular differences at baseline rather than differential adaptations to IET. These pre-existing differences that contribute to elevated peripheral vascular resistance (Bunsawat et al. 2021; Koenen et al. 2021) may be responsible for the limited magnitude of dBP and MAP reduction achievable through short-term IET for individuals with high-BMI.

The absence of between-group differences in all other haemodynamic variables measured suggests that the attenuated BP response in the high-BMI group was not driven by divergent adaptations in cardiac output or stroke volume. Moreover, despite the well-documented autonomic differences between normal and high-BMI individuals at rest (Espinoza-Salinas et al. 2021), contributing to increases in BP (Valensi 2021), there were no significant between-group differences were observed in any HRV or BRS outcome following IET. Interestingly, both groups demonstrated broadly comparable autonomic adaptations irrespective of BMI category.

Notwithstanding the attenuated BP responsiveness, both BMI groups experienced clinically meaningful improvements in BP following IET compared to the control group. Of note, sBP reductions were similar between groups (normal BMI: -10.7 [-13.4, -7.9] mmHg; high BMI: -9.0 [-11.7, -6.2] mmHg) and both exceeded the magnitude of sBP reductions reported in a recent large-scale network meta-analysis by Edwards et al. (2023); aerobic exercise training (~ 4–7 mmHg), resistance training (~ 4–5 mm Hg), concurrent training (~ 6 mmHg), and high-intensity interval-based approaches (~ 4–5 mm Hg). This suggests that while adiposity may moderate the magnitude of benefit, individuals with high BMI can still obtain therapeutic effects from IET, although they may require additional strategies to optimise dBP reductions, such as combining IET with dietary modification (Ben-Dov et al. 2000; Bacon et al. 2004), or more prolonged IET training durations (Pourmotahari et al. 2025; O’Driscoll et al. 2022).

### Strengths and limitations

This study has several strengths, including a relatively large sample size for IET research and an even mix of female and male participants. The dual use of ANCOVA and change-score analyses provides a robust assessment of between-group differences. However, some limitations should be acknowledged. The study employed a 4-week intervention, which, while sufficient to induce measurable reductions in BP, may not fully capture the longer-term adaptations needed to overcome the physiological constraints associated with adiposity (Pourmotahari et al. 2025). Additionally, BMI is a crude indicator of body composition and does not distinguish between fat mass, visceral adiposity, and lean tissue, each of which may differentially influence vascular and autonomic responses (Koenen et al. 2021). Future work should incorporate direct measures of body composition and explore whether modifying IET variables (e.g., intensity, frequency, contraction mode) can enhance responsiveness in higher-BMI individuals. Notably, the high-BMI group was significantly taller than the normal-BMI group. As BMI is indexed to height squared, systematic differences in stature may influence group classification and potentially confound interpretation of adiposity-related effects. Moreover, limb length, particularly femur length, may influence mechanical loading and haemodynamic responses during lower-body wall-squat IET, given its impact on joint angles and torque requirements at a fixed position. Future research should incorporate direct measures of body composition and anthropometry, including limb segment length, to better isolate adiposity-related effects and determine whether morphological characteristics modify responsiveness to IET. Lastly, we acknowledge that grouping overweight and obese individuals into a high-BMI group may limit the granularity of conclusions. Future research with larger samples should examine whether BP responsiveness to IET differs between overweight and obese class I individuals specifically.

## Conclusion

In summary, this study demonstrates that BMI moderates the BP-lowering effects of IET, with higher BMI individuals experiencing attenuated reductions in dBP and MAP relative to individuals with normal BMI. While both groups benefited from training, these findings highlight the importance of considering BMI when prescribing IET for BP management and suggest that adjunctive or extended interventions may be needed to optimise outcomes in individuals with high BMI.

All authors contributed to the study conception and design. Material preparation, data collection and analysis were performed by Harry Swift and Jim Wiles. The study design was developed by Harry Swift, Jim Wiles and Chris Farmer. The first draft of the manuscript was written by Harry Swift and all authors commented on previous versions of the manuscript. All authors read and approved the final manuscript.

## Abbreviations

ANCOVA: Analysis of covariance
BL: Baseline
BMI: Body mass index
BP: Blood pressure
BRS: Baroreflex sensitivity
CI: Cardiac index
CI: Confidence interval
CO: Cardiac output
CON: Control
dBP: Diastolic blood pressure
ECG: Electrocardiogram
HF: High-frequency
HR: Heart rate
HRpeak: Peak heart rate
HRV: Heart rate variability
IET: Isometric exercise training
IL-6: Interleukin-6
LF: Low-frequency
MAP: Mean arterial pressure
MCP-1: Monocyte chemoattractant protein-1
MET: Metabolic equivalent of task
NO: Nitric oxide
PSD-RRI: Power spectral density of R–R intervals
Q̇: Cardiac output
Q̇I: Cardiac index
sBP: Systolic blood pressure
SD: Standard deviation
SI: Stroke volume index
SV: Stroke volume
SVi: Stroke volume index
TFM: Task force monitor
TNF-α: Tumour necrosis factor-alpha
TPR: Total peripheral resistance
TPRi: Total peripheral resistance index
WHO: World health organization
